# Clinical Experience of Timing Treatment in Newborns with Spinal Muscular Atrophy: A Call for Standardized Screening Practices in Italy

**DOI:** 10.3390/ijns12010016

**Published:** 2026-03-09

**Authors:** Ilaria Bitetti, Rosa Iannaccone, Giovanna Margiotta, Antonio Varone

**Affiliations:** 1 Pediatric Neurology, Santobono-Pausilipon Children’s Hospital, 80129 Naples, Italy; a.varone@santobonopausilipon.it; 2 Department of Pharmacy, Santobono-Pausilipon Children’s Hospital, 80129 Naples, Italy; r.iannaccone@santobonopausilipon.it (R.I.); g.margiotta@santobonopausilipon.it (G.M.)

**Keywords:** spinal muscular atrophy (SMA), newborn screening, *SMN1*/*SMN2* gene, genetic screening

## Abstract

Spinal muscular atrophy (SMA) is a rare neuromuscular disorder causing progressive muscle weakness. Severe SMA forms are typically observed up to six months postnatally. Disease-modifying therapies provide significant benefits, making newborn screening (NBS) essential for timely diagnosis and treatment initiation. The NBS programme evaluated infants born between April 2023 and October 2024 in the Campania region, Italy. DNA was amplified to detect homozygous deletion of the SMN1 gene by RT-PCR and *SMN2* copy number using multiplex ligation-dependent probe amplification. Following treatment, motor functions were assessed using CHOP-INTEND and Bayley III scales. Among 62,801 infants screened for SMA, thirteen (11 females, 2 males) tested positive. The distribution of *SMN2* copy numbers was as follows: eight patients had two copies, one patient had three, and four patients had four copies. One year after treatment, motor outcome data were available for four of the eight patients with two *SMN2* copies. Among these patients, one achieved the milestones of walking without support, and three were standing with support. At 24 months, three of these patients were walking independently. Pre-symptomatic treatment markedly improves motor function development. This underscores the urgent need for large-scale newborn screening to prevent diagnostic delays and ensure timely, effective therapy. Validated care protocols must be established to facilitate early diagnosis and intervention.

## 1. Introduction

Spinal muscular atrophy (SMA) is a rare neuromuscular condition primarily caused by homozygous deletions in the survival motor neuron (*SMN*) type 1 gene encoding for the SMN protein. Common symptoms of SMA include severe muscle weakness with the risk of impaired ambulation and respiratory and swallowing difficulties [1,2].

Although deletions in the *SMN1* gene are responsible for SMA in 95% of cases, the homologous *SMN2* gene copy number, which causes the production of a truncated unstable protein, highly impacts the severity and clinical outcome of SMA patients. Individuals with SMA are categorised into five subtypes (from 0 to 4), which reflect disease severity based on the age of disease presentation—from prenatal to adulthood—and the level of motor function. With an estimated incidence of 1 in 10,000 births, SMA is globally recognised as the second most prominent cause of infant mortality after cystic fibrosis [3,4]. In Italy, recent nationwide data estimate a prevalence of 2.12 per 100,000 inhabitants, corresponding to 1255 patients currently followed in specialised centres. The distribution by clinical subtype includes 284 patients with SMA type I, 470 with type II, 467 with type III, and 15 with type IV. Notably, approximately 85% of patients are receiving disease-modifying therapies [5].

Newborn screening (NBS) programmes, based on genetic testing, represent an unparalleled approach for the timely identification of infants at risk of SMA. The first-tier methodology of NBS commonly relies on quantitative PCR (qPCR) on DNA isolated from dried blood spots (DBSs) after birth to detect homozygous deletion of the *SMN1* gene. Deletion screen-positive cases undergo further confirmatory second-tier testing through advanced PCR techniques to confirm *SMN1* deletion and test for *SMN2* copy number [6].

Several pilot NBS programmes are ongoing worldwide, yet a few have been implemented [7,8]. The absence of a national implementation of the newborn screening programme in Italy is not attributable to a single barrier but rather to a complex interplay of factors, including the need to standardise molecular diagnostic techniques, improve testing for *SMN2* gene variants, establish robust cost-effectiveness data in certain regions, and address regional disparities in healthcare infrastructure and priorities, all despite increasing evidence supporting the programme’s benefits. For instance, although NBS practice is present on a regional scale in Italy, mandatory implementation at the national level remains to be established [8,9,10]. As underscored by the SMA working group of American and European healthcare providers, reducing the time to diagnose and identify SMA cases before the occurrence of symptoms ensures timely treatment and better clinical outcomes [11]. Approved novel disease-modifying therapies (DMTs) have been game-changing for infants affected by SMA. These innovative therapies include nusinersen, an antisense oligonucleotide; onasemnogene abeparvovec, a gene therapy-based product; and risdiplam, an orally bioavailable *SMN2* splicing modifier that increases systemic SMN protein levels by improving exon 7 inclusion [12]. These therapies all aim to increase the expression of or restore the functional production of the SMN protein.

Several single-arm multicentre trials have demonstrated that initiating treatment at around 1 month of age in pre-symptomatic newborns with DMTs results in the achievement of most motor milestones [13]. Furthermore, comparative observational studies confirmed that pre-symptomatic newborns, compared with symptomatic or untreated cohorts, achieve independent motor competencies [14,15,16,17]. These reports confirm that NBS is paramount, as delays in diagnosing and initiating treatment for newborns with SMA can significantly worsen long-term outcomes [18].

Compelling clinical results from advanced therapies for SMA have revolutionised the perspectives of both families and clinicians, confirming the need to streamline and standardise NBS procedures worldwide. Nonetheless, NBS programmes remain heterogeneous and inconsistent across different countries [19]. Several challenges remain to be addressed, such as a lack of multidisciplinary partnerships across clinical centres, treatment initiation delays, and the harmonisation of standard NBS practices [6].

The primary aim of this work is to describe the implementation and clinical experiences of NBS programmes for SMA in the Campania region, Italy, and to report the timing of diagnosis and treatment initiation in infants identified through NBS. This could provide valuable real-world insights from a regional healthcare setting, highlighting the importance of timely intervention and the need for validated care protocols to support early diagnosis in infants with SMA.

## 2. Materials and Methods

### 2.1. NBS Programme and Participants

This is a descriptive, observational study reporting real-world clinical data from a regional NBS programme for SMA. The primary objective is to describe the implementation and clinical feasibility of a regional NBS programme, and to report the timing of diagnosis and treatment initiation in infants identified through NBS. Secondary objectives include the descriptive evaluation of early motor and neurodevelopmental outcomes following early treatment, with the goal of informing the development of standardised, evidence-based care pathways for infants diagnosed with SMA through NBS.

As part of a regional initiative, infants born between April 2023 and October 2024 in the Campania region of Italy were screened for SMA [20]. The project involved all birth centres and neonatal intensive care units (NICUs) across the region. It was coordinated by A.O.R.N. Santobono-Pausilipon in collaboration with CEINGE—Advanced Biotechnologies Franco Salvatore in Naples and was funded by the Campania Region and Novartis Gene Therapies.

The molecular test for SMA was performed alongside mandatory newborn screenings. Upon parental consent, a single heel-prick blood sample was collected from newborns before hospital discharge between the second and third day after birth. The sample was sent to the CEINGE—Advanced Biotechnologies Franco Salvatore laboratory for processing. Genomic DNA was extracted from the dried blood spot (DBS) and exon 7 of the *SMN1* gene was amplified by real-time polymerase chain reaction (RT-PCR) on a semiautomated EONIS™ platform (PerkinElmer Italy S.p.A., Milan, Italy). In the case of a positive screening result, the family was contacted and invited to the referral centre for SMA care at A.O.R.N. Santobono—Neurology to begin the clinical pathway. Diagnostic confirmation and determination of the number of *SMN2* copies were performed using multiplex ligation-dependent probe amplification (MLPA) (SALSA MLPA Probemix p021 (B1-0619), MRC Holland, Amsterdam, The Netherlands). Results are available within 48–72 h, and in case of SMA, the newborn is immediately referred to the Santobono reference centre. A paediatric neurologist, together with a psychologist, consult to communicate the diagnosis and discuss therapeutic options. Blood samples are collected for MLPA testing. Standard laboratory analyses including transaminases and complete blood count, and assessment of anti-AAV9 antibodies are performed. The therapeutic choice depends on the number of *SMN2* copies, the clinical picture, and parental preference. For infants with up to three *SMN2* copies and a body weight >2.6 kg, gene therapy may be indicated, provided there is no positivity for anti-AAV9 antibodies. In children with four or more *SMN2* copies, the recommended treatments are nusinersen or risdiplam. For infants with two *SMN2* copies or those already showing symptoms of SMA, treatment should be initiated as early as possible, ideally within the first 20 days of life. No standardised national protocol was available in Italy to guide treatment selection for infants diagnosed with SMA through NBS at the time of analysis. Therefore, treatment decisions followed a structured clinical pathway based on *SMN2* copy number, baseline clinical status, eligibility criteria for each disease-modifying therapy, and shared decision-making with families.

All positive subjects were treated with one of the three approved DMTs for SMA, such as onasemnogene abeparvovec (Zolgensma^®^, Novartis—Durham, NC, USA), nusinersen (Spinraza^®^, Biogen—Cambridge, MA, USA), or risdiplam (Evrysdi^®^, Roche—Basel, Svizzera). Before treatment, a compound muscle action potential (CMAP) test of the ulnar nerve was recorded at baseline. Patients were categorised as asymptomatic, paucisymptomatic, or symptomatic based on neurological examination and standardised motor assessment, in accordance with current best practice recommendations [11]. Asymptomatic patients showed no clinical signs suggestive of SMA. Paucisymptomatic patients presented early or subtle neuromuscular findings potentially suggestive of SMA but not yet clearly attributable to overt disease, such as hyporeflexia, mild or borderline hypotonia, or motor function and overall psychomotor development scores, defined by the Children’s Hospital of Philadelphia Infant Test of Neuromuscular Disorder (CHOP-INTEND), at the lower limit of normal for age (approximately 40 points in neonates). Symptomatic patients were defined by the presence of unequivocal clinical signs of SMA, including absent deep tendon reflexes, frank hypotonia, and age-inappropriate pathological CHOP-INTEND scores.

**Figure 1:**
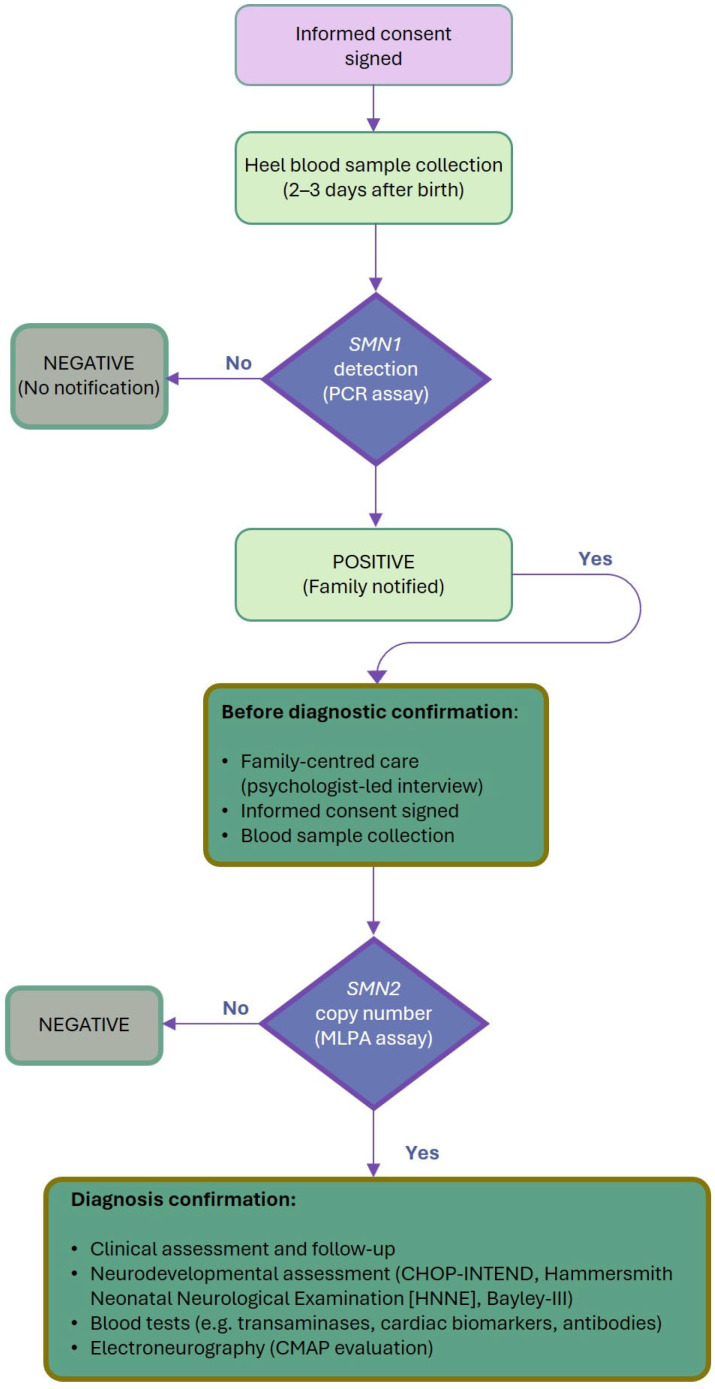
Workflow of the NBS pathway activated in the Campania region for SMA diagnosis. The flowchart illustrates the diagnostic screening and therapeutic management process for SMA-positive infants. CMAP, compound muscle action potential; MLPA, multiplex ligation-dependent probe amplification; SMA, spinal muscular atrophy; SMN, survival motor neuron.

At pre-defined follow-up, neurological examination of the infant included standardised assessments of motor function and overall psychomotor development by CHOP-INTEND scale and the Bayley III scale of Infant and Toddler Development—third edition. The flowchart in Figure 1 provides a comprehensive overview of the workflow of the NBS pathway activated in the Campania region.

### 2.2. Ethics Approval

The study was reported to the Ethics Committee at the Santobono-Pausilipon Children’s Hospital, Naples, Italy. The families participating in the study provided written informed consent.

### 2.3. Data Description

Demographic characteristics and clinical examinations were presented using frequency and percentage. Data from the Bayley III scale were reported as composite and percentile scores.

## 3. Results

### 3.1. Demographics and Clinical Features

From April 2023 to October 2024, 62,801 newborns were screened for SMA in the Campania region. Thirteen newborns (11 females, 2 males) tested positive for recessive deletions in exons 7 and 8 of the *SMN1* gene through the NBS programme via MLPA. Genetic screening confirmed that all parental couples were carriers of the exon 7 and/or 8 deletion in the SMN1 gene. Baseline demographic and clinical characteristics of the entire cohort are reported in Table 1.

#### 3.1.1. Patients with Two *SMN2* Copies

Within the cohort, eight patients (61%) had two copies of *SMN2*, corresponding to the severe SMA phenotype. Five of these patients were identified presymptomatically and treated within the first 22 days of life. Two patients were paucisymptomatic, and one patient was symptomatic (Table 1).

#### 3.1.2. Patients with ≥3 *SMN2* Copies

Five patients carried three or more copies of *SMN2*, a genotype typically associated with a milder disease course. Specifically, one (8%) had three copies of *SMN2*, and four (31%) had four copies of *SMN2*. All were asymptomatic at diagnosis and received early treatment following NBS (Table 1).

### 3.2. Compound Muscle Action Potential (CMAP) Testing and Therapy

During the first clinical evaluation, 10 out of 13 patients were asymptomatic. Two patients were paucisymptomatic at first visit: patient 9 (hyporeflexia), and patient 11 (first visit at 8 days old). The day before starting therapy (fifteenth day of life), patient 11 developed marked hypotonia and absent deep tendon reflexes, fulfilling the predefined criteria for symptomatic SMA. Accordingly, the patient was reclassified as symptomatic before therapy initiation. Patient 4 was symptomatic. CMAP amplitudes were within the normal range for all asymptomatic patients except for patient 4 (Table 2).

Patient 4 displayed severe hypotonia, tongue fasciculations, brisk tendon reflexes and poor motility. The median age of patients starting therapy with DMTs following confirmation of SMA diagnosis was 17 days. Follow-up visits were scheduled as part of their care programme. Of the eight patients with two *SMN2* copies, seven (1, 2, 3, 4, 11, 12 and 13) received onasemnogene abeparvovec gene therapy, and underwent weekly clinical neurological assessments and blood tests during the first month post-infusion, followed by assessments every two weeks until the third month, and then every 1–2 months throughout the first year. Follow-up was conducted every 3–6 months in the second year. Assessments in symptomatic patient 4 included polysomnography, which did not suggest sleep disorders. Patient 6, carrying three *SMN2* copies, was treated with nusinersen for the first 2 months due to an elevated anti-AAV9 antibodies titre. The patient then received the loading dose. After the 4th dose, the anti-AAV9 antibodies titre was decreased to the eligibility limit for gene therapy, allowing treatment with onasemnogene abeparvovec. Check-ups were conducted every two weeks until the fourth month, followed by every four months, with intermediate clinical follow-ups if needed. Five patients (5, 7, 8 and 10), with four copies of *SMN2*, and patient 9, with two copies of *SMN2*, were treated with risdiplam and were evaluated at follow-up every 2–6 months during the first two years. None of the patients required non-invasive ventilation (NIV).

### 3.3. Motor and Cognitive Ability Assessments

**Figure 2:**
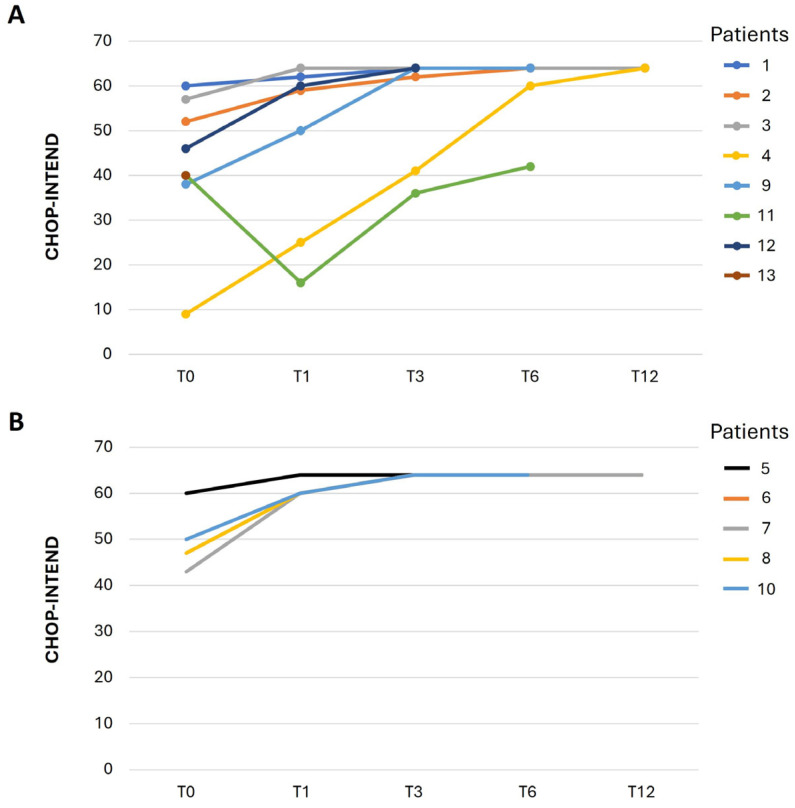
CHOP-INTEND scale assessment in newborns with SMA. The assessment was conducted at baseline (T0) at the time of diagnosis and after 1 (T1), 3 (T3), 6 (T6) and 12 (T12) months following therapy. CHOP-INTEND, Children’s Hospital of Philadelphia Infant Test of Neuromuscular Disorder; SMA, spinal muscular atrophy. (**A**) Patients with two *SMN2* copies. (**B**) Patients with ≥3 *SMN2* copies.

The group of thirteen infants was evaluated using the CHOP-INTEND test and motor milestone assessments at the time of diagnosis (time 0) and through a series of follow-ups at one (time 1), three (time 3), six (time 6), and twelve months (time 12) following therapy (Figure 2; Table 3).

This test ranges from 0 to 64, with higher scores suggesting amelioration of motor function. Patients 1, 2, 3, 4, 9, 11, 12, and 13, with two copies of *SMN2*, improved motor functions. After one year of treatment, patients 1, 2, 3 and 4 showed optimal motor developmental milestones: patients 2, 3 and 4 could stand with support, and patient 1 progressed to walking without support. At the time of the present study, patients 1, 2 and 3 walked independently (follow-up at 24 months), while patients 9, 11, 12 and 13 needed to complete the follow-up. Patient 9 scored 38 at baseline and displayed no head control and at 6 months is now sitting with support (Table 3). Patients 5, 6, 7, 8 and 10, with three or four copies of SMN2, improved motor function from baseline, reaching a score of 60 after three months of therapy. At one year follow-up, patients 5, 6 and 7 were walking without support, while patient 10, after 6 months of treatment, was sitting without support (Table 3).

The Bayley III scale was used to evaluate cognitive, linguistic, and motor development at six (time 6) and twelve months (time 12) from baseline (time 0) (Table 4).

Developmental delay is generally considered to be significant if the Bayley score is under 70 points, mild between 70 and 85, and indicative of a better outcome if ≥ 100 [16,21]. In line with the CHOP-INTEND test, for patients with two copies of *SMN2*, cognitive language, and motor scores remained stable or improved after 6 and 12 months of therapy in patient 1, 2, 3 and 4. Follow-up is ongoing for patients 9, 11, 12 and 13. Patients 9 and 11 did not show any score improvement in all three scales after six months of therapy. Patients 12 and 13 were evaluated at baseline only, and good functioning was suggested in all three developmental areas. Concerning patients with three or four copies of *SMN2*, cognitive language, and motor scores improved for patient 5, while they remained stable in patient 6. After 6 months of therapy, the cognitive and language scales of patient 7 displayed an improvement whereas motor development remained poor. The cognitive, language and motor scales of patient 10 suggested mild impairment. Patient 8 did not improve in cognitive and language scores after six months of therapy, showing increased scores only for motor domain.

## 4. Discussion

The identification of symptomatic and pre-symptomatic patients with SMA requires urgent standardisation of NBS practice to stratify the risk of neurodevelopmental decline and start targeted therapy, potentially attenuating disease progression. This study describes a real-world regional experience of NBS for SMA, highlighting the feasibility of early diagnosis, timely treatment initiation, and the clinical relevance of standardised care pathways for infants identified through NBS.

In this report, seven patients with two *SMN2* copies at risk of developing severe SMA were treated pre-symptomatically. Patients with two *SMN2* copies represent the subgroup at highest risk for severe SMA. In the present cohort, early identification through NBS allowed treatment initiation before or shortly after symptom onset in most cases. In this subgroup, motor outcomes observed during follow-up reported benefits of early or presymptomatic treatment, including progressive improvement in motor function and acquisition of major milestones in patients with longer follow-up [6]. However, motor outcomes were not uniform across individuals, likely due to differences in clinical status at the time of treatment initiation. In such cases, treatment can stabilise disease progression and prevent further deterioration rather than fully restore lost motor function. Thus, variability in baseline clinical status and follow-up duration should be considered when interpreting these findings. Patients with three or more *SMN2* copies are typically associated with a milder disease course. In our cohort, all patients in this subgroup were asymptomatic at diagnosis and received early treatment following NBS. During follow-up, these patients showed a generally progressive motor development, although the short observation period limits conclusions.

Gene therapy is often the first-line option, as it is a one-time treatment and adverse effects in newborns are generally minimal due to the immaturity of the immune system. As a result, patients achieved optimal motor milestones, including walking independently for three of them. At present, there is no universally accepted algorithm guiding therapeutic decision-making for patients diagnosed with SMA [22]. The three currently approved disease-modifying therapies have each demonstrated consistent stabilisation or improvement of disease progression, although interindividual variability in treatment response has been observed [23,24,25]. No clinical trial has conclusively shown superior efficacy or tolerability among the available agents. Consequently, treatment selection remains influenced by multiple factors, including contraindications, route and frequency of administration, safety profile and duration of therapy, family preferences, patient age, and clinical phenotype. Moreover, a scientific consensus is still lacking regarding the optimal initial treatment strategy and the potential role of combination approaches to achieve additional long-term benefits. The greatest clinical benefit has been associated with presymptomatic and early initiation of treatment, underscoring the critical role of NBS programmes in enabling timely diagnosis and intervention.

The treatment choice is made jointly with the family. If daily oral administration is challenging for caregivers, or if the infant presents feeding intolerance or frequent regurgitation, treatment with nusinersen may be preferred. Conversely, if parents refuse the lumbar puncture required for nusinersen administration, risdiplam may be selected instead. Previous evidence documents that despite heterogeneous disease evolution in pre-symptomatic patients with two *SMN2* copies, the majority treated with onasemnogene abeparvovec reached relevant motor milestones [23]. Although the number of patients of this phenotype reported here is limited, the findings unequivocally reinforce that prompt therapy provides significant benefits before overt disease manifestation. Moreover, four patients with four *SMN2* copies described in this report were treated with risdiplam, with good achievements at 12 months (stand with support and walking without support) for two of them, although complete response remains to be evaluated during follow-up visits. Increasing clinical findings demonstrated the favourable safety/efficacy profile of risdiplam and positive recovery of motor skills [26]. The preliminary results from the RAINBOWFISH study confirmed that, regardless of the *SMN2* copy number, infants treated pre-symptomatically with risdiplam up to 12 months were able to achieve sitting, standing and walking motor milestones [27].

Notably, the *SMN2* copy number significantly impacts the clinical phenotype. Among patients with SMA type I or who were presymptomatic, those with three *SMN2* copies correlate with milder manifestations of neurodegeneration, delayed onset of ambulation difficulties, and more prolonged survival compared to individuals with two *SMN2* copies [28]. While the relationship between *SMN2* copy number and therapeutic efficacy remains uncertain, close follow-up monitoring of pre-symptomatic patients is critical. Early recognition of hyporeflexia, muscle weakness, and delayed milestone achievement can facilitate timely treatment and beneficial prognosis. Thus, NBS is a vital intervention pathway that can alleviate the burden of SMA, enhance the patient’s quality of life, and ultimately lower healthcare costs [29]. As identified in this study, prompt genetic screening and clinical evaluation through CMAP testing and neurodevelopmental assessment scales allow for early diagnosis and maximise the intervention window.

In clinical assessments, the adoption of comprehensive neurodevelopmental scales is paramount not only to identify high-risk children but also to investigate the impact of the therapeutic intervention and monitor rehabilitative care. The landscape of NBS management is still vague due to the absence of standardised laboratory methods for screening and follow-up tests. There are no established guidelines to prevent treatment delays, standardised clinical endpoints, or effective multidisciplinary care coordination for the routine assessment of SMA patients [19,30]. In addition, the traditional SMA classification based solely on clinical milestones has become increasingly inadequate to guide prognosis and treatment choices. In this evolving context, the introduction of NBS may also influence future prevalence estimates by enabling the early identification of milder SMA phenotypes that were previously underdiagnosed, redefining the epidemiological landscape of the disease. An updated classification combining diagnostic timing (presymptomatic or symptomatic), *SMN2* copy number, and baseline clinical status is also needed to support individualised therapeutic decisions [31].

Recent recommendations for NBS best practice highlight the importance of clinically characterising SMA phenotypes and emphasise the cooperation required between laboratory and healthcare services [11]. Essential standard operating procedures should prioritise prompt initial follow-up visits, access to efficient treatment options, and *SMN2* copy number identification during diagnostic confirmatory testing. A prominent challenge, extensively discussed in prior studies and reiterated by this work, pertains to implementing consistent care protocols and approved guidelines across the medical community [8,32]. Addressing this issue is essential to ensure timely diagnoses and optimise disease outcomes. Moreover, the support to families and caregivers, the core component of the management of SMA, is strongly required [11]. The psychosocial and emotional load of parents should be addressed through expert counselling [33]. In addition to emotional reassurance, specialised care should foster active engagement and education via informative materials and verbal communication. This approach aims to create co-management of care in partnerships with families to guide shared decision-making and prevent treatment delays in infants identified through NBS. As new emergent therapies improve life expectancy for patients with SMA, this collaborative care model should anticipate their social and cognitive needs during the transition to adulthood. A comprehensive health and social care ecosystem should adapt to the evolving needs of these patients and prioritise access to tailored educational programmes and resources to maximise the quality of life of these individuals [34].

This study has some limitations. First, the cohort size is small and reflects the clinical experience of a single regional screening hub, which does not allow for generalizable conclusions. Moreover, not all patients had completed the follow-up at the time of this report, preventing a complete longitudinal assessment of outcomes for the entire cohort. Third, given the absence of a comparator group and the descriptive nature of the analysis, the findings should be interpreted as exploratory. The heterogeneity of treatments represents an additional limitation of this study. Patients received different disease-modifying therapies based on *SMN2* copy number, clinical status, eligibility criteria, and family preference, and one patient underwent sequential therapy. This variability precludes any comparison between treatments. Therefore, this work should not be interpreted as evidence of treatment equivalence, but rather as a reflection of real-world clinical practice. Finally, the occurrence of false negatives or potential cases missed during the screening period were not systematically investigated. Further studies are deemed necessary to investigate the long-term efficacy of DMTs for SMA patients.

## Figures and Tables

**Table 1:** Demographics and characteristics of the cohort identified by NBS.

Patient	Gender	Gestational Age	Number of *SMN2* Copies	Weight (kg)	Age at First Treatment (Days)	Baseline Symptomatic Status
1	F	39	2	4.18	22	asymptomatic
2	F	33	2	3.50	17	asymptomatic
3	F	39	2	4.00	20	asymptomatic
4	F	38	2	3.70	17	symptomatic
5	M	39	4	3.96	40	asymptomatic
6	F	39	3	4.30	11	asymptomatic
7	F	40	4	4.00	9 *	asymptomatic
8	F	39	4	3.00	13	asymptomatic
9	F	39	2	3.50	19	paucisymptomatic
10	F	39	4	3.50	17	asymptomatic
11	M	38	2	3.60	16	paucisymptomatic
12	F	40	2	3.60	13	asymptomatic
13	F	39	2	3.10	13	asymptomatic

F, female; M, male; SMN, survival motor neuron. * In patient 7, early treatment initiation (9 days of life) reflected rapid drug availability and prompt parental consent.

**Table 2:** Compound muscle action potential (CMAP) test. Following clinical assessment, the patients were assigned to DMTs. DMTs, disease-modifying therapies.

Patient	CMAP (Baseline)	Medication
1	>5 mV	onasemnogene abeparvovec
2	>5 mV	onasemnogene abeparvovec
3	>5 mV	onasemnogene abeparvovec
4	<1 mV	onasemnogene abeparvovec
5	>5 mV	risdiplam
6	>5 mV	nusinersen *
7	>5 mV	risdiplam
8	>5 mV	risdiplam
9	>5 mV	risdiplam
10	>5 mV	risdiplam
11	>5 mV	onasemnogene abeparvovec
12	>5 mV	onasemnogene abeparvovec
13	>5 mV	onasemnogene abeparvovec

* Patient 6 switched to onasemnogene abeparvovec after 2 months (following the nusinersen loading dose).

**Table 3:** Motor milestones assessment in newborns with SMA. The assessment was conducted at baseline (T0) at the time of diagnosis and after 1 (T1), 3 (T3), 6 (T6) and 12 (T12) months following therapy. SMA, spinal muscular atrophy. Assessments at (T1) for patient 7 and at (T3), (T6) and (T12) for patients 6 and 7 are not available yet. Missing data indicate assessments not yet performed because the scheduled follow-up time point had not been reached at the time of data analysis.

Patient	Milestone T0	Milestone T1	Milestone T3	Milestone T6	Milestone T 12	Milestone T 24
1	no head control	head control	rolling	sitting without support	walking without support	walking
2	no head control	head control	head control	sitting without support	stand with support	walking
3	no head control	head control	rolling	sitting without support	stand with support	walking
4	no head control	no head control	head control	rolling, sitting with support	stand with support	—
5	no head control	head control	rolling	sitting without support	walking without support	—
6	no head control	head control	rolling	sitting without support	walking without support	—
7	no head control	head control	rolling	sitting without support	walking without support	—
8	no head control	head control	head control	sitting without support	—	—
9	no head control	head control	head control	sitting with support	—	—
10	no head control	head control	rolling	sitting without support	—	—
11	no head control	no head control	head control	head control	—	—
12	no head control	head control	rolling	—	—	—
13	no head control	—	—	—	—	—

**Table 4:** Bayley III scale assessment in newborns with SMA. The assessment, including cognitive, language and motor evaluation, was conducted at baseline (T0) at the time of diagnosis and after 6 (T6) and 12 (T12) months following therapy. Values are expressed as composite scores and percentile scores in brackets. SMA, spinal muscular atrophy. Missing data indicate assessments not yet performed because the scheduled follow-up time point had not been reached at the time of data analysis.

	Cognitive			Language			Motor		
Patient	T0	T6	T12	T0	T6	T12	T0	T6	T12
1	90 (25)	105 (63)	120 (91)	83 (13)	94 (34)	112 (79)	85 (16)	112 (79)	118 (88)
2	90 (25)	85 (16)	85 (16)	89 (23)	83 (13)	94 (34)	97 (42)	91 (27)	82 (12)
3	95 (37)	95 (37)	95 (37)	100 (50)	100 (50)	94 (34)	103 (58)	107 (68)	85 (16)
4	100 (50)	105 (63)	120 (91)	94 (34)	106 (66)	95 (37)	67 (1)	61 (0.5)	65 (1)
5	100 (50)	85 (16)	120 (91)	103 (58)	94 (34)	112 (79)	107 (68)	82 (12)	118 (88)
6	100 (50)	95 (37)	95 (37)	100 (50)	100 (50)	94 (34)	110 (75)	107 (68)	85 (16)
7	100 (50)	105 (63)	—	94 (34)	106 (66)	—	100 (50)	91 (27)	—
8	90 (25)	85 (16)	—	83 (13)	83 (13)	—	85 (16)	112(79)	—
9	85 (16)	85 (16)	—	83 (13)	83 (13)	—	67 (1)	82 (12)	—
10	100 (50)	105 (63)	—	103 (58)	106 (66)	—	107 (68)	112 (79)	—
11	85 (16)	85 (16)	—	94 (34)	86 (13)	—	67 (1)	61 (0.5)	—
12	100 (37)	—	—	100 (58)	—	—	107 (68)	—	—
13	100 (50)	—	—	103 (58)	—	—	100 (50)	—	—

## Data Availability

The authors confirm that the data supporting the findings of this study are available within the article.

## Abbreviations

The following abbreviations are used in this manuscript:

AAV9	Adeno-Associated Virus serotype 9
CEINGE	Centro di Ingegneria Genetica e Biotecnologie Avanzate “Franco Salvatore”
CHOP-INTEND	Children’s Hospital of Philadelphia Infant Test of Neuromuscular Disorders
CMAP	Compound Muscle Action Potential
DBS	Dried Blood Spot
DMTs	Disease-Modifying Therapies
MLPA	Multiplex Ligation-Dependent Probe Amplification
NBS	Newborn Screening
NIV	Non-Invasive Ventilation
qPCR	Quantitative Polymerase Chain Reaction
RT-PCR	Real-Time Polymerase Chain Reaction
SMA	Spinal Muscular Atrophy
SMN	Survival Motor Neuron
